# Supporting Obesity Prevention in Statewide Quality Rating and Improvement Systems: A Review of State Standards

**DOI:** 10.5888/pcd14.160518

**Published:** 2017-12-07

**Authors:** Nora Ann Geary, Carrie Ann Dooyema, Meredith Ann Reynolds

**Affiliations:** 1 Division of Nutrition, Physical Activity, and Obesity, Centers for Disease Control and Prevention, Atlanta, Georgia

## Abstract

**Introduction:**

A quality rating and improvement system (QRIS) is a fundamental component of most states’ early care and education infrastructures. States can use a QRIS to set standards that define high-quality care and award child care providers with a quality rating designation based on how well they meet these standards. The objective of this review was to describe the extent to which states’ QRIS standards include obesity prevention content.

**Methods:**

We collected publicly available data on states’ QRIS standards. We compared states’ QRIS standards with 47 high-impact obesity prevention components in *Caring for Our Children: National Health and Safety Performance Standards; Guidelines for Early Care and Education Programs, 3rd Edition,* and 6 additional topics based on the Centers for Disease Control and Prevention’s *Spectrum of Opportunities for Obesity Prevention in the Early Care and Education Setting*.

**Results:**

Thirty-eight states operated a state-wide QRIS in early 2015. Of those, 27 states’ QRIS included obesity prevention standards; 20 states had at least one QRIS standard that aligned with the high-impact obesity prevention components, and 21 states had at least one QRIS standard that aligned with at least one of the 6 additional topics. QRIS standards related to the physical activity high-impact obesity prevention components were the most common, followed by components for screen time, nutrition, and infant feeding.

**Conclusion:**

The high proportion of states operating a QRIS that included obesity prevention standards, combined with the widespread use of QRISs among states, suggests that a QRIS is a viable way to embed obesity prevention standards into state early care and education systems.

## Introduction

The Centers for Disease Control and Prevention (CDC) identifies Early Care and Education (ECE) as a priority setting for public health obesity prevention efforts (1). Every state public health department (including the District of Columbia), as a grantee of CDC’s State Public Health Actions, requires activities specific to the ECE setting (2). CDC supports state grantees to embed nationally recommended obesity prevention standards into components of their ECE systems by using a guiding framework, *Spectrum of Opportunities for Obesity Prevention in the Early Care and Education Setting (Spectrum of Opportunities)* (3). The *Spectrum of Opportunities* outlines common mechanisms through which states can support ECE facilities to achieve recommended standards for obesity prevention. One component of this framework is a quality rating and improvement system (QRIS).

A QRIS is a core component of most states’ ECE infrastructures and is relatively new; most states began operating a QRIS by 2012 (4). QRIS is one approach to assess and improve quality in child care settings. Through a QRIS, states establish standards that define gradations of quality of care and award a quality rating designation to participating ECE programs based on how well they meet these standards. Many states use a star rating system, with more stars indicating higher quality. Some states use licensing regulations as the basis of their lowest quality rating designation. States’ QRIS standards typically cover the following categories: professional development, qualifications, training, and accreditation; parent and family involvement; learning environment; licensing compliance and status; staff compensation; administrative policies and procedures; financial management; and program evaluation (5). It is generally accepted that quality child care can result in improved child outcomes (6–10), and research on QRISs and childhood outcomes demonstrates that highly rated programs have a positive effect on children’s development (11,12). However, little is known about how states use their QRIS as a mechanism to encourage healthy nutrition, physical activity, and other obesity prevention strategies in child care, all of which are important components of healthy development for young children (13). The objective of our review was to describe the extent to which states’ QRIS standards include obesity prevention content.

## Methods

We used a stepwise process to determine which states had a statewide QRIS in operation, first using an online public database, the QRIS Compendium (14). Second, because state participation in QRISs is rapidly expanding, we conducted independent web searches for each state that was reported to not have a QRIS to confirm that no state QRIS existed. Third, we collected information on QRIS standards from official state websites. For the 4 states without QRIS standards posted on official websites, we contacted the QRIS operating agency. We collected data from January through April 2015.

### Inclusion and exclusion criteria

Of the 50 US states and the District of Columbia, 38 states had a statewide QRIS operating during early 2015 with standards available for review. Twelve states and the District of Columbia were excluded from the sample for the following reasons: we were unable to determine whether the state had a QRIS in operation (Wyoming, South Dakota); the state was precluded from operating a QRIS through legislative action (Missouri); the QRIS was operating at a local level (California, Florida); or the QRIS was in a developmental or piloting phase (Alaska, Connecticut, District of Columbia, Hawaii, Kansas, Louisiana, Virginia, West Virginia). These criteria yielded a final sample of 38 states that had a QRIS with publicly available standards published between 2007 and 2015.

### Review methodology

Each state’s QRIS standards were read by 2 researchers (N.A.G. and C.A.D.) in their entirety and compared with the 47 high-impact obesity prevention components described in *Caring for Our Children: National Health and Safety Performance Standards; Guidelines for Early Care and Education Programs, 3rd Edition *(15). These 47 components (hereinafter referred to as PCO/CFOC) were identified as high impact through an expert review process (15) and are categorized into 4 domains: infant feeding (n = 11 components), nutrition (n = 21 components), physical activity (n = 11 components), and screen time (n = 4 components). The PCO/CFOC components have been used since 2010 by the National Resource Center for Health and Safety in Child Care and Early Education for an annual assessment of states’ licensing regulations (16). Several studies used these components and a methodology similar to the one described here to assess the presence of obesity prevention content in state child care licensing and regulations (17–21).

If a state’s QRIS standard contained language that matched either fully or partially with one or more of the 47 PCO/CFOC components, it was recorded as present. All discrepancies in recording were resolved through discussion and consensus. To ensure that no standards were overlooked, one reviewer (N.A.G.) read each state’s standards a second time. Data were collected in an Excel database.

We also reviewed each state’s QRIS standards for 6 topics beyond the PCO/CFOC components. These topics were based on the *Spectrum of Opportunities* and are complementary strategies to PCO/CFOC components. These include 1) participation or adherence to the Child and Adult Care Food Program (CACFP), 2) a facility-level self-assessment specific to nutrition, physical activity, screen time, breastfeeding, or obesity prevention (eg, a nutritional environment assessment tool), 3) action planning tools for obesity prevention–related areas (eg, an action plan for active physical play), 4) professional development training for obesity prevention–related topics (eg, a requirement that providers complete), 5) technical assistance from professionals with subject matter expertise relevant to obesity prevention (eg, a consultation from trained dieticians), and 6) family-engagement resources or activities related to obesity prevention (eg, a family resource center with nutrition information).

## Results

About one-quarter of states’ QRIS (11 of 38) had no standards related to obesity prevention. Of the 27 QRISs that included obesity prevention standards, 20 had at least one standard that aligned with a PCO/CFOC component, and 21 had at least one standard that aligned with at least one of the 6 *Spectrum of Opportunities* topics (Table 1).

**Table 1:** Summary of Information on Quality Rating and Improvement Systems With at Least One Standard That Aligns With PCO/CFOC Components^a^ and/or *Spectrum of Opportunities* Components^b^, by State (n = 27)^c^, 2010–2015

State	Name of QRIS (Date of Publication of Standards)	No. of PCO/CFOC Components^a^ Present in QRIS Standards	No. of *Spectrum of Opportunities* Components^b^ Present in QRIS Standards
Arkansas	Better Beginnings (2010)	1	2
Colorado	Colorado Shines (2014)	2	3
Georgia	Quality Rated (2012)	0	2
Idaho	Steps to Quality (unknown)	0	3
Iowa	Iowa’s Quality Rating System (2011)	0	3
Indiana	Paths to Quality (2008)	4	0
Massachusetts	Massachusetts QRIS (2010)	1	1
Maryland	Maryland Excels (2014)	8	2
Maine	Quality for ME (unknown)	1	0
Michigan	Great Start to Quality (2013)	1	2
Minnesota	Parent Aware (2013)	0	2
Montana	Best Beginnings STARS to Quality (2014)	3	2
North Dakota	Bright and Early North Dakota (2012)	1	2
Nebraska	Step Up to Quality (unknown)	0	4
New Jersey	Grow NJKids (2014)	1	3
New Mexico	FOCUS (2015)	4	2
Nevada	Nevada Silver State Stars QRIS (2014)	4	4
New York	QUALITY Stars NY (2014)	10	2
Oklahoma	Oklahoma Reaching for the Stars (unknown)	3	0
Oregon	Oregon QRIS (unknown)	8	0
Pennsylvania	Pennsylvania Keystone STARS (2014–2015)	0	1
Rhode Island	BrightStars (2013)	1	0
South Carolina	ABC Quality (2012–2013)	15	3
Texas	Texas Rising Star (2012)	4	0
Utah	Care About Childcare (unknown)	10	2
Washington	Early Achievers (unknown)	0	1
Wisconsin	YoungStar (2014)	2	2

a Forty-seven obesity prevention components, referred to as PCO/CFOC, are described in *Caring for Our Children: National Health and Safety Performance Standards; Guidelines for Early Care and Education Programs, 3rd Edition *(15).

b Components (n = 6) are based on the Centers for Disease Control and Prevention’s *Spectrum of Opportunities for Obesity Prevention in the Early Care and Education Setting *(3) and are complementary strategies to PCO/CFOC components.

c Thirty-eight states had a Quality Rating and Improvement System with publicly available standards published between 2007 and 2015; of these, 11 states had no Quality Rating and Improvement Systems standards aligning with PCO/CFOC components or the 6 additional *Spectrum of Opportunities* (3) components: Alabama, Arizona, Delaware, Illinois, Kentucky, Mississippi, North Carolina, New Hampshire, Ohio, Tennessee, Vermont. Twelve states and the District of Columbia were excluded from the review for various reasons: unable to determine whether the state had a QRIS in operation (Wyoming, South Dakota); precluded from operating QRIS through legislative action (Missouri); QRIS operating at a local level (California, Florida); and QRIS in a developmental or piloting phase (Alaska, Connecticut, District of Columbia, Hawaii, Kansas, Louisiana, Virginia, West Virginia).

### PCO/CFOC components

Six states’ QRIS included at least one standard that aligned with at least one PCO/CFOC component for infant feeding. Two of the 11 PCO/CFOC components for infant feeding were included in states’ standards: feeding of breast milk for young infants (IA1, 5 states) and holding infants while bottle feeding (IB3, 1 state) (Table 2). The remaining 9 PCO/CFOC infant feeding components were not present in states’ QRIS. For example, no state had standards for feeding infants on cue, allowing infants to stop feedings, developing a plan along with a parent or guardian for introducing age-appropriate foods, serving fruit juice to infants, or serving whole fruits to older infants (Table 2).

**Table 2:** States That Have at Least One Standard That Aligns With PCO/CFOC Components^a^, by Standard, 2010–2015^b^

Standard	State	No. of States
**Infant feeding (11 items) **		
**IA1. **Encourage/support breastfeeding by onsite arrangements for moms to breastfeed	Montana, New Jersey, New York, Nevada, Utah	5
**IA2. **Serve milk or formula until at least 12 months of age	0	0
**IB1. **Feed infants on cue	0	0
**IB2. **Do not feed infants beyond satiety/allow infant to stop the feeding	0	0
**IB3. **Hold infants while bottle feeding	Texas	1
**IC1. **Develop a plan for introducing age-appropriate solid foods in consultation with the child’s parent/guardian and primary care provider	0	0
**IC2. **Introduce age-appropriate solid foods no sooner than 4 months of age, and preferably around 6 months of age	0	0
**IC3. **Introduce breastfed infants gradually to iron-fortified foods no sooner than 4 months, and preferably at 6 months	0	0
**ID1. **Do not feed an infant formula mixed with cereal, juice, or other foods	0	0
**ID2. **Serve whole fruits, mashed or pureed, for infants aged 7 months to 1 year	0	0
**ID3. **Serve no fruit juice to children younger than 12 months	0	0
**Nutrition (21 items)**		
**NA1**. Limit oils by choosing monounsaturated fats and polyunsaturated fats and avoiding *trans* fats, saturated fats, and fried foods	Maryland, South Carolina	2
**NA2**. Serve meats and/or beans, avoiding fried meats	South Carolina	1
**NA3.** Serve other milk equivalent products (yogurt, cottage cheese) using low-fat variants to children 2 years or older	0	0
**NA4**. Serve whole milk to children aged 12 to 24 months who are not on human milk, or serve reduced-fat milk to those at risk for hypercholesterolemia or obesity	Nevada	1
**NA5**. Serve skim or 1% milk to children aged 2 years or older	Nevada, South Carolina	2
**NB1**. Serve whole-grain breads, cereals, and pastas	Maryland, South Carolina	2
**NB2**. Serve vegetables (dark green, orange, deep yellow, and root, such as potatoes and viandas)	Maryland, Oregon, South Carolina, Utah	4
**NB3**. Serve fruits of several varieties, especially whole	Maryland, Oregon, South Carolina, Utah	4
**NC1. **Only 100% juice, no added sweeteners	0	0
**NC2.** Offer juice (100%) only during meal times	0	0
**NC3**. Serve no more than 4–6 ounces of juice per day to children aged 1–6 years	South Carolina	1
**NC4.** Serve no more than 8–12 ounces of juice per day to children aged 7–12 years	NA^c^	NA^c^
**ND1**. Make water available both inside and outside	Utah	1
**NE1.** Teach children appropriate portion sizes by using plates, bowls, and cups that are developmentally appropriate to nutritional needs	0	0
**NE2**. Adults eating meals with children eat items that meet standards	Montana, Utah	2
**NF1**. Serve small-sized, age-appropriate portions	Texas	1
**NF2**. Permit children to have 1 or more additional servings of nutritious foods that are low in fat, sugar, and sodium as needed to meet the caloric needs of the child; teach children who require limited portions about portion size; monitor their portions	Texas	1
**NG1**. Limit salt by avoiding salty foods (chips, pretzels)	Maryland	1
**NG2**. Avoid sugar, including concentrated sweets (candy, sodas, sweetened drinks, fruit nectars, flavored milk)	Maryland, South Carolina	2
**NH1**. Do not force or bribe children to eat	Indiana, Montana, Oregon, South Carolina, Texas	5
**NH2. **Do not use food as a reward or punishment	0	0
**Physical activity (11 items) **		
**PA1**. Provide adequate space, both inside and outside play	Colorado, Indiana, Massachusetts, Maine, North Dakota, Oklahoma, Oregon, Rhode Island, Utah	9
**PA2. **Provide orientation and annual training opportunities for caregivers/teachers to learn age-appropriate gross motor activities and games that promote physical activity	Arkansas	1
**PA3**. Develop written policies on the promotion of physical activity and the removal of potential barriers to physical activity participation	South Carolina	1
**PA4**. Require caregivers/teachers to promote children’s active play and participate in children’s active games at times when they can safely do so	Oregon, South Carolina	2
**PA5. **Do not withhold active play from children who misbehave	South Carolina	1
**PC1**. From birth to 6 years, provide 2 or 3 occasions daily of active play outdoors, weather permitting	Indiana, Michigan, New York, Oklahoma, South Carolina, Utah	6
**PC2**. For toddlers, provide 60–90 minutes per 8-hour day of moderate to vigorous physical activity	New York, Wisconsin	2
**PC3**. For preschoolers, provide 90–120 minutes per 8-hour day for moderate to vigorous physical activity	New York, Wisconsin	2
**PD1**. From birth to 6 years, provide 2 or more daily structured or adult-led activities or games that promote movement	Arkansas, Colorado, Nevada, New York	4
**PE1**. Daily supervised tummy time for infants	New York	1
**PE2**. Use infant equipment (swings, stationary centers, seats, bouncers) only for short periods of time if at all	South Carolina	1
**Screen time (4 items)**		
**PB1**. Do not utilize media (television, video, or DVD) viewing and computer with children younger than 2 years	Maryland, New Mexico, New York, Oklahoma, Oregon, South Carolina, Utah	7
**PB2**. Limit total media time for children aged 2 years or older to no more than 30 minutes per week	New Mexico, New York, Oregon, Utah	4
**PB3**. Use screen media with children age 2 years and older only for educational purposes or physical activity	Indiana, Maryland, New Mexico, New York, Oregon, Utah	6
**PB4**. Do not utilize television, video, or DVD viewing during meal or snack time	New Mexico, New York	2

a Forty-seven obesity prevention components, referred to as PCO/CFOC, are described in *Caring for Our Children: National Health and Safety Performance Standards; Guidelines for Early Care and Education Programs, 3rd Edition *(15). The standards listed in this table have been abbreviated.

b The letter–number combinations (eg, 1A1) correspond to the letter–number combinations used in the coding system of the National Resource Center (16).

c Not applicable to children aged 0 to 5 years.

Eight states’ QRIS included standards that aligned with at least one PCO/CFOC component for nutrition (Table 2). Fifteen of 21 PCO/CFOC components were addressed. The most common components were prohibiting the use of force or bribery to get children to eat (NH1, 5 states); serving fruits and vegetables (NB2 and NB3, 4 states); limiting oils and fats (NA1, 2 states); serving low-fat milk to children 2 years or older (NA5, 2 states); serving whole grains (NB1, 2 states); avoiding sugar (NG2, 2 states); and setting nutritional requirements for adults who eat meals with children (NE2, 2 states). Five PCO/CFOC components for nutrition were not included in any states’ QRIS standards. For example, no QRIS standards addressed serving low-fat milk alternatives (eg, yogurt, cottage cheese), teaching children about portion sizes, or prohibiting the use of food as a reward or punishment.

Fifteen states’ QRIS included standards that aligned with at least one PCO/CFOC component for physical activity. All 11 PCO/CFOC components for physical activity were addressed (Table 2). The most common PCO/CFOC component present in states’ QRIS pertained to providing adequate space for inside and outside play (PA1, 9 states), providing 2 or 3 occasions of active play outdoors daily (PC1, 6 states), and encouraging caregivers to lead structured play (eg, activities or games) (PD1, 4 states). Fewer states’ QRIS specified the amount of time toddlers and preschoolers should be moderately to vigorously active (PC2 and PC3, 2 states each), or had standards that required caregivers to promote children’s active play and participate in children’s active games (PA4, 2 states). PCO/CFOC components for infant physical activity were less common (PE1 and PE2, 1 state each), as were standards for training providers in topics related to physical activity (PA2, 1 state), developing written policies to promote physical activity (PA3, 1 state), and prohibiting withholding active play from children who misbehave (PA5, 1 state).

Eight states’ QRIS included standards that aligned with at least one PCO/CFOC component for screen time (Table 2). Seven states’ QRIS addressed not using screen time for children aged 2 or younger (PB1). Six states addressed allowing screen time only for educational or physical activity purposes for children aged 2 years or older (PB3), and 4 states had a standard for limiting screen time for children aged 2 years or older to no more than 30 minutes per week (PB2). Only 2 states’ QRIS had standards prohibiting media use during meal or snack time (PB4) (Table 2).

### *Spectrum of Opportunities* standards 

Twenty-one states’ QRIS had at least one standard aligning with the 6 *Spectrum of Opportunities* components, which went beyond the PCO/CFOC components (Table 3). Twelve states’ QRIS standards referenced participating in or adhering to CACFP meal pattern requirements. Six states’ QRIS standards included a facility-level self-assessment related to obesity prevention, of which 5 addressed multiple topic areas (eg, nutrition and physical activity). Four states had a QRIS standard for facility-level action planning focused on at least one obesity prevention strategy area (eg, physical activity). Ten states had professional development trainings for nutrition and/or physical activity as stand-alone QRIS standards, of which nutrition was the most common topic addressed. Six states’ QRIS standards included technical assistance from a health consultant, child care health consultant, and/or nutritionist. Ten states had QRIS standards for engaging families through various strategies, such as providing education about nutrition and/or physical activity.

**Table 3:** States That Have at Least One Standard That Aligns With *Spectrum of Opportunities* Components^a^, by State (n = 21), 2010–2015

State	*Spectrum of Opportunities* Component^a^					
	CACFP	Facility-Level Assessment Tools	Facility-Level Action Planning	Professional Development	Technical Assistance	Family Engagement
Arkansas	—	—	—	Nutrition, Physical activity	—	Nutrition, Physical activity
Colorado	—	—	—	Nutrition	Child care health consultant	Nutrition, Physical activity
Georgia	—	Nutrition, Physical activity	Nutrition, Physical activity	—	—	—
Idaho	—	Nutrition, Physical activity	Nutrition, Physical activity	—	Child care health consultant	—
Iowa	Yes	—	—	Nutrition	Child care health consultant	—
Maryland	Yes	—	—	—	—	Nutrition, Physical activity
Massachusetts	—	—	—	—	Child care health consultant	—
Michigan	Yes	—	—	—	—	Nutrition
Minnesota	Yes	—	—	Nutrition, Physical activity, Obesity prevention	—	—
Montana	Yes	—	—	Nutrition	—	—
North Dakota	Yes	Nutrition, physical activity	—	—	—	—
Nebraska	Yes	Nutrition, Physical activity, Breastfeeding, Screen time	—	Nutrition, Physical activity, Breastfeeding, Screen time	—	Nutrition, Obesity prevention
New Jersey	Yes	Nutrition, Physical activity, Breastfeeding, Screen time	—	—	—	Nutrition, Obesity prevention
New Mexico	—	—	Nutrition, Physical activity, Obesity prevention	Nutrition	—	—
Nevada	Yes	Nutrition	Nutrition, Physical activity	—	Child care health consultant, Nutritionist	—
New York	Yes	—	—	Obesity prevention	—	—
Pennsylvania	—	—	—	—	—	Nutrition, Physical activity
South Carolina	Yes	—	—	Nutrition, Physical activity	—	Physical activity
Utah	—	—	—	—	Child care health consultant	Nutrition, Physical activity
Washington	—	—	—	—	—	Nutrition, Physical activity
Wisconsin	Yes	—	—	Nutrition	—	—

Abbreviations: —, does not have component; CACFP, Child and Adult Care Food Program.

a Components (n = 6) are based on the Centers for Disease Control and Prevention’s *Spectrum of Opportunities for Obesity Prevention in the Early Care and Education Setting *(3) and are complementary strategies to PCO/CFOC components. Forty-seven obesity prevention components, referred to as PCO/CFOC, are described in *Caring for Our Children: National Health and Safety Performance Standards; Guidelines for Early Care and Education Programs, 3rd Edition *(15).

### Other standards related to obesity prevention

Although we examined only standards that aligned with the 47 PCO/CFOC components and the 6 *Spectrum of Opportunities* topics, we found that 17 states had other standards related to obesity prevention or promoting healthy lifestyles (Arkansas, Colorado, Delaware, Indiana, Massachusetts, Maryland, Michigan, Montana, Nebraska, New Jersey, New Mexico, Nevada, New York, Oregon, South Carolina, Texas, and Utah). For example, Colorado had a QRIS standard that awards points to programs that have a garden and serve fruits and/or vegetables from it for children to taste, and New York had a QRIS standard that awarded points to programs that adopt a formal obesity prevention program.

## Discussion

Our review found obesity prevention–related standards in 27 states’ QRIS, 20 of which related to at least one PCO/CFOC component, and 21 of which related to a *Spectrum of Opportunities* topic. Twenty-two states had fewer than 5 standards related to a PCO/CFOC component, suggesting that states have the potential to embed more obesity prevention standards into their QRIS. PCO/CFOC components related to physical activity were most common in states’ QRIS standards, followed by screen time, nutrition, and infant feeding.

Most of the 27 states had QRIS standards related to the PCO/CFOC physical activity components; the most common QRIS standard was related to providing physical space for both inside and outside play. Few states had QRIS standards promoting physical activity (eg, number of minutes per day). Because physical activity is important not only in obesity prevention but also in the cognitive and physical development of young children (22), states could improve in this area.

Few states had QRIS standards addressing PCO/CFOC infant feeding components. These findings are consistent with those in a review of child care licensing and regulations for best practices in infant feeding (23). States could explore opportunities to embed infant feeding standards into their QRIS as a strategy to fill gaps or to build on existing standards.

Adherence to, or participation in, CACFP was a common standard. This is an encouraging finding because adhering to CACFP guidelines ensures that children are served nutritious meals and snacks. Furthermore, some evidence shows that programs that participate in CACFP have practices that align with several PCO/CFOC components, such as offering whole-grain foods and fruits and vegetables and having providers eat the same foods that are offered to children (24,25).

Much of the research on obesity prevention in states’ ECE systems focuses on child care licensing and regulations and their practical implications (17–21). Our review also has practical implications, especially for advancing ECE obesity prevention in a state QRIS. First, using our review as a baseline assessment, states can monitor progress in QRISs, just as the National Resource Center reviews obesity prevention content in states’ child care licensing and regulations (16,26). Second, states interested in establishing a QRIS can examine our data to identify viable options for their own state and to identify peer states for consultation. Third, because a QRIS can build on other state policy and environmental levers, such as child care licensing and regulations, readers can use our data in conjunction with other reports to get a more complete understanding of their state ECE systems’ inclusion of PCO/CFOC standards (26,27). Finally, states revising their QRIS standards could consider using standards in *Caring for Our Children* as a way to include national best practices for obesity prevention.

Our review has several strengths. Several reports identify ECE as a key setting for early childhood obesity prevention (eg, Scientific Report of the 2015 Dietary Guidelines Advisory Committee [28], Surgeon General’s Vision for a Healthy and Fit Nation 2010 [29]), and our review provides insight into how states have included obesity prevention–related standards into their QRIS. Our review builds on previous work (30,31) on obesity prevention in QRISs by demonstrating what has occurred in several states and how a QRIS can be used to address obesity prevention in ECE. It also serves as the first detailed baseline report of states’ work in QRISs as a mechanism for obesity prevention. As more states establish a QRIS, states could consider strengthening the language of standards to bring them closer to fully meeting PCO/CFOC components. For example, a state’s QRIS standard that partially meets a PCO/CFOC component says, “breastfeeding is encouraged and the environment and program policies are designed to support this.” Adding language about “making arrangements for mothers to feed their children comfortably on-site” would bring it closer to fully meeting the PCO/CFOC component (IA1). Because the methodology used in our review aligns with the monitoring of child care licensing and regulations, state public health departments, early learning stakeholders, and directors of state QRISs can use our findings in conjunction with other reports to get a more complete picture of how well their state’s ECE system supports obesity prevention (26,27). Finally, our methodology could be used by other researchers interested in exploring the inclusion of other *Caring for Our Children* standards in state QRISs (eg, childhood mental health standards). *Caring for Our Children* has more than 600 standards with thousands of components on various health and safety topics, including infectious disease, positive behavior management, sun safety, oral health, and use of consultants in early childhood mental health and early education.

Our review has several limitations. We relied on publicly available QRIS materials from states’ websites, and it is possible that the materials were not current. Second, a state’s licensing regulations were not factored into the review even when the regulations were used as the basis of the lowest quality rating designation for the state (32). Third, the methodology relied on subjective interpretations of states’ QRIS standards. Although we were careful in using a well-known framework for obesity prevention in child care settings and adapting a published methodology, reviewers relied on the written text of states’ QRIS standards as the sole basis to determine whether each obesity prevention component was included (15). For example, if a standard referenced a “physical activity checklist,” coders gave credit to the state for having a facility-level assessment that addressed obesity prevention. However, the content of facility-level assessment and action planning tools included in QRIS standards were not reviewed. This limitation extends to standards related to professional development and technical assistance.

QRISs have grown in popularity in the United States during the last decade, partially as a result of the Race to the Top Early Learning Challenge (33) and other actions, such as the reauthorization of the Child Care Development Block Grant. With continued support of quality improvement initiatives for child care, states may continue to strengthen their QRIS. Although participation in a QRIS is currently voluntary for ECE providers in most states, with the exception that some states require ECE providers that receive state child care subsidy funds to participate, states are increasingly providing incentives and using other strategies to increase participation (30). For these reasons, a QRIS can be considered as a potential lever in a state’s ECE system to embed obesity prevention standards and may provide a systematic way to improve obesity prevention policies and practices in many ECE settings.

Although we suggest QRIS is a viable option for embedding obesity prevention into a state’s ECE system, we also note that various factors influence whether a state chooses to pursue the establishment of a QRIS or another mechanism, such as child care licensing and regulations, to improve ECE environments. Moreover, what is viable in one state may not be viable in another. A single strategy alone, such as a QRIS, is unlikely to improve the quality of ECE environments. Rather, a series of approaches that build on each other, such as those outlined in CDC’s *Spectrum of Opportunities*, may be needed to achieve widespread change. As our review shows, a QRIS is one strategy that states are pursuing as part of a layered approach to set standards for higher-quality child care; however, long-term health outcomes and the prevention of obesity are influenced by many factors that extend beyond the child care setting (34).

CDC continues to provide support to states in their efforts to address obesity prevention in ECE through policy, systems, and environmental change, and a QRIS is one of several mechanisms states can pursue. State agencies can use findings from our review to better understand QRISs and opportunities to support obesity prevention in ECE.

## References

[1] Early care and education. Atlanta (GA): US Department of Health and Human Services, Centers for Disease Control and Prevention, National Center for Chronic Disease Prevention and Health Promotion, Division of Nutrition, Physical Activity and Obesity; 2015. https://www.cdc.gov/obesity/strategies/childcareece.html. Accessed August 15, 2016.

[2] State public health actions to prevent and control diabetes, heart disease, obesity and associated risk factors and promote school health. Atlanta (GA): US Department of Health and Human Services, Centers for Disease Control and Prevention, National Center for Chronic Disease Prevention and Health Promotion; 2015. http://www.cdc.gov/chronicdisease/about/state-public-health-actions.htm. Accessed August 15, 2016.

[3] The spectrum of opportunities for obesity prevention in the early care and education setting, CDC technical assistance briefing document. Atlanta (GA): US Department of Health and Human Services, Centers for Disease Control and Prevention, National Center for Chronic Disease Prevention and Health Promotion, Division of Nutrition, Physical Activity and Obesity; 2015. https://www.cdc.gov/obesity/downloads/Spectrum-of-Opportunities-for-Obesity-Prevention-in-Early-Care-and-Education-Setting_TAbriefing.pdf. Accessed August 15, 2016.

[4] Top ten questions about QRIS. QRIS compendium: a catalog and comparison of quality rating and improvement systems (QRIS); 2017. www.qriscompendium.org/top-ten/question-1. Accessed January 4, 2017.

[5] Overview of the QRIS resource guide. Washington (DC): US Department of Health and Human Services, Administration for Children & Families Office of Child Care, National Center on Child Care Quality Improvement; 2015. https://qrisguide.acf.hhs.gov/files/QRIS_Resource_Guide_2015.pdf. Accessed August 30, 2016.

[6] Muennig P , Schweinhart L , Montie J , Neidell M . Effects of a prekindergarten educational intervention on adult health: 37-year follow-up results of a randomized controlled trial. Am J Public Health 2009;99(8):1431–7. 10.2105/AJPH.2008.148353 19542034 PMC2707464

[7] Reynolds AJ , Temple JA , Ou SR . School-based early intervention and child well-being in the Chicago Longitudinal Study. Child Welfare 2003;82(5):633–56. 14524429

[8] Camilli G , Vargas S , Ryan S , Barnett WS . Meta-analysis of the effects of early education interventions on cognitive and social development. Teach Coll Rec 2010;112(3):579–620. http://www.tcrecord.org/content.asp?contentid=15440

[9] Hahn RA , Barnett WS , Knopf JA , Truman BI , Johnson RL , Fielding JE , Early childhood education to promote health equity: a Community Guide systematic review. J Public Health Manag Pract 2016;22(5):E1–8. 10.1097/PHH.0000000000000378 26672406 PMC4969181

[10] National Institute of Child Health and Human Development Early Child Care Research Network. Early child care and children’s development prior to school entry: results from the NICHD Study of Early Child Care. Am Educ Res J 2002;39(1):133–64. 10.3102/00028312039001133

[11] Sabol TJ , Pianta RC . Validating Virginia’s quality rating and improvement system among state-funded pre-kindergarten programs. Early Child Res Q 2015;30:183–98. 10.1016/j.ecresq.2014.03.004

[12] Tout K , Cleveland J , Weilin L , Starr R , Soli M , Bultinck, E . The parent aware evaluation: initial validation report. Minneapolis (MN): Child Trends; 2016. http://s3.amazonaws.com/Omnera/VerV/s3finder/38/pdf/Parent%20Aware%20Validation%20Report_Final.pdf. Accessed August 15, 2016.

[13] Tandon PS , Tovar A , Jayasuriya AT , Welker E , Schober DJ , Copeland K , The relationship between physical activity and diet and young children’s cognitive development: a systematic review. Prev Med Rep 2016;3:379–90. 10.1016/j.pmedr.2016.04.003 27419040 PMC4929214

[14] QRIS Compendium. A catalog and comparison of quality rating and improvement systems (QRIS); 2017. http://qriscompendium.org/. Accessed August 15, 2016.

[15] Preventing childhood obesity in early care and education: selected standards from caring for our children: national health and safety performance standards; guidelines for early care and education programs, 3rd Edition. Washington (DC): Academy of Pediatrics, American Public Health Association, and National Resource Center for Health and Safety in Child Care and Early Education; 2012. http://cfoc.nrckids.org/StandardView/SpcCol/Preventing_Childhood_Obesity. Accessed August, 15, 2016.

[16] Achieving a state of healthy weight: a national assessment of obesity prevention terminology in child care regulations 2010. Aurora (CO): National Resource Center for Health and Safety in Child Care and Early Education, University of Colorado Denver; 2011. http://nrckids.org/default/assets/File/Products/ASHW/regulations_report_2010.pdf. Accessed September 6, 2016.

[17] Benjamin SE , Cradock A , Walker EM , Slining M , Gillman MW . Obesity prevention in child care: a review of U.S. state regulations. BMC Public Health 2008;8(1):188. 10.1186/1471-2458-8-188 18513424 PMC2438347

[18] Story M , Kaphingst KM , French S . The role of child care settings in obesity prevention. Future Child 2006;16(1):143–68. 10.1353/foc.2006.0010 16532662

[19] Kaphingst KM , Story M . Child care as an untapped setting for obesity prevention: state child care licensing regulations related to nutrition, physical activity, and media use for preschool-aged children in the United States. Prev Chronic Dis 2009;6(1):A11. 19080017 PMC2644584

[20] Benjamin SE , Taveras EM , Cradock AL , Walker EM , Slining MM , Gillman MW . State and regional variation in regulations related to feeding infants in child care. Pediatrics 2009;124(1):e104–11. 10.1542/peds.2008-3668 19564255 PMC3049909

[21] Slining MM , Neelon SE , Duffey KJ . A review of state regulations to promote infant physical activity in child care. Int J Behav Nutr Phys Act 2014;11(1):139. 10.1186/s12966-014-0139-3 25416613 PMC4247659

[22] Tandon PS , Tovar A , Jayasuriya AT , Welker E , Schober DJ , Copeland K , The relationship between physical activity and diet and young children’s cognitive development: a systematic review. Prev Med Rep 2016;3:379–90. 10.1016/j.pmedr.2016.04.003 27419040 PMC4929214

[23] Benjamin SE , Taveras EM , Cradock AL , Walker EM , Slining MM , Gillman MW . State and regional variation in regulations related to feeding infants in child care. Pediatrics 2009;124(1):e104–11. 10.1542/peds.2008-3668 19564255 PMC3049909

[24] Liu ST , Graffagino CL , Leser KA , Trombetta AL , Pirie PL . Obesity prevention practices and policies in child care settings enrolled and not enrolled in the Child and Adult Care Food Program. Matern Child Health J 2016;20(9):1933–9. 10.1007/s10995-016-2007-z 27112556

[25] Blaine RE , Davison KK , Hesketh K , Taveras EM , Gillman MW , Benjamin Neelon SE . Child care provider adherence to infant and toddler feeding recommendations: findings from the Baby Nutrition and Physical Activity Self-Assessment for Child Care (Baby NAP SACC) study. Childhood obesity 2015;11(3):304–13. 25918873 10.1089/chi.2014.0099 PMC4485887

[26] National Resource Center for Health and Safety in Child Care and Early Education. Achieving a state of healthy weight. 2017. http://nrckids.org/index.cfm/products/achieving-a-state-of-healthy-weight1/. Accessed June 22, 2017.

[27] Summary of obesity prevention standards in state quality rating and improvement systems (QRIS) and licensing regulations. Washington (DC): Nemours Children’s Health System; 2016. https://d3knp61p33sjvn.cloudfront.net/2016/04/SummaryofObesityPreventionLicensingRegulationsandQRISStandards_040416.pdf. Accessed June 22, 2017.

[28] Scientific report of the 2015 Dietary Guidelines Advisory Committee: advisory report to the Secretary of Health and Human Services and the Secretary of Agriculture. US Department of Agriculture and US Department of Health and Human Services; 2015. https://health.gov/dietaryguidelines/2015-scientific-report/PDFs/Scientific-Report-of-the-2015-Dietary-Guidelines-Advisory-Committee.pdf. Accessed September 22, 2016.

[29] Surgeon General’s vision for a healthy and fit nation 2010. Rockville (MD): US Department of Health and Human Services; 2010. http://www.ncbi.nlm.nih.gov/books/NBK44660/pdf/Bookshelf_NBK44660.pdf. Accessed September 6, 2016.

[30] State quality rating and improvement systems: strategies to support achievement of healthy eating and physical activity practices in early care and education settings. Washington (DC): Nemours Children’s Health System; 2016. https://d3knp61p33sjvn.cloudfront.net/2016/07/State_QRIS_Strategies_to_Support_Achievement_of_Healthy_Eating_and_Physical_Activity_Practice_in_ECE_Settings-June2016.pdf. Accessed September 1, 2016.

[31] State efforts to address obesity prevention in child care quality rating and improvement systems. Atlanta (GA): Altarum Institute; 2012. http://altarum.org/sites/default/files/uploaded-related-files/QRIS-Report-22Feb12-FIN_0.pdf. Accessed September 1, 2016.

[32] QRIS resource guide. Fairfax (VA): US Department of Health and Human Services, Office of Child Care (TA Network); 2015. https://qrisguide.acf.hhs.gov/index.cfm?do=section&sid=3. Accessed June 22, 2017.

[33] Zellman GL , Perlman M , Le V , Setodji CM . Assessing the validity of the Qualistar early learning quality rating and improvement system as a tool for improving child-care quality. Santa Monica (CA): Rand Corporation; 2008. http://www.rand.org/content/dam/rand/pubs/monographs/2008/RAND_MG650.pdf. Accessed June 22, 2017.

[34] Shilder D , Iruka I , Dichter H , Mathias D . Quality rating and improvement systems: stakeholder theories of change and models of practice. 2015. http://qrisnetwork.org/sites/all/files/resources/2016-02-10%2009%3A21/QRIS%203.0%20Report%20V11%202016.2.5%20FINAL.pdf. Accessed June 20, 2017.

